# Estrogen receptor 1 signaling in hepatic stellate cells designates resistance to liver fibrosis

**DOI:** 10.1038/s41421-025-00783-3

**Published:** 2025-04-15

**Authors:** Tianhao Li, Gang Wang, Han Zhao, Fuhai Liu, Zhangyuzi Deng, Dingbao Chen, Xin Zhou, Ying Cao, Wei Fu, Haoyue Zhang, Jing Yang

**Affiliations:** 1 School of Life Sciences, Peking University Third Hospital Cancer Center, Center for Life Sciences, State Key Laboratory of Membrane Biology, IDG/McGovern Institute for Brain Research, Peking University, Beijing, China; 2 Department of Hepatobiliary, Peking University People’s Hospital, Beijing, China; 3 Institute of Molecular Physiology, Shenzhen Bay Laboratory, Shenzhen, Guangdong China; 4 Department of General Surgery, Peking University Third Hospital, Beijing, China; 5 Peking Union Medical College Hospital, Beijing, China

**Keywords:** Mechanisms of disease, Hormone receptors

Dear Editor,

The prevalence and severity of liver fibrosis are higher in men than in premenopausal women, while the disease tends to worsen in postmenopausal women. However, the pathophysiological mechanism underlying such clinical manifestations remains incompletely understood. In this study, we show that sex hormone depletion in adult female mice exaggerates liver fibrosis, while estradiol replacement in castrated male mice is sufficient to mitigate the disease. Transcriptomic profiling and immunohistochemistry then demonstrate that both human and mouse hepatic stellate cells (HSCs), the primary cell type responsible for extracellular fibrous depositions, predominantly express the estrogen receptor 1 (ESR1). The HSC-specific genetic deletion of *ESR1* markedly promotes liver fibrosis. Moreover, chromatin immunoprecipitation sequencing (ChIP-seq) and in vitro manipulations suggest that ESR1 can regulate the expression of key fibrosis-related genes in HSCs. These results have elucidated a critical aspect of ESR1 signaling in the sexual dimorphism of liver fibrosis.

Liver fibrosis is a pathological process with the excessive deposition of extracellular matrix proteins, particularly collagens, in this vital organ. The disease often accompanies chronic damage to hepatocytes, e.g., viral hepatitis, biliary obstruction, alcoholic liver disease, or metabolic dysfunction-associated steatotic liver disease (MASLD, formerly known as nonalcoholic fatty liver disease). While the formation of fibrotic scars represents an integrative step of tissue repair, it can lead to the impairment of normal liver functions and eventually progress to more severe complications, including cirrhosis and hepatocellular carcinoma^1^.

HSCs are the primary cell type involved in the production of extracellular matrix proteins during liver fibrosis^2^. Under the healthy condition, HSCs are in a quiescent state characterized by the stellate morphology and the storage of vitamin A (i.e., retinol) in their lipid droplets. Upon pathological stimulation, HSCs are triggered into a proliferative, fibrogenic state with the loss of stellate morphology, decrease of vitamin A storage, and enhanced release of extracellular matrix proteins. Activated HSCs designate the severity of fibrosis through their complex crosstalk with Kupffer cells, lymphocytes, sinusoidal endothelial cells, and other cell types in the liver. Therefore, comprehensive knowledge of the fibrogenic response of HSCs is essential for more effective therapeutic strategies against this debilitating disease.

Clinical evidence has indicated that the prevalence and severity of liver fibrosis and its related hepatic or extra-hepatic outcomes exhibit significant sex differences^3–7^. For instance, in patients with MASLD, men were at a higher risk of more severe fibrosis than premenopausal women, while postmenopausal women had a similar disease severity to men^8^. These observations suggested that estrogens may confer resistance to liver fibrosis. However, the mechanism of the potential estrogen modulation of HSCs remains incompletely understood.

As the entry point, we exploited bilateral ovariectomy in adult C57BL/6 wild-type female mice to mimic menopausal hormone depletion (Supplementary Fig. S1a). Compared to the mice receiving sham surgery, the ovariectomized mice fed with the normal chow diet (NCD) did not exhibit liver steatosis or fibrosis in the timeframe of our experimental setup (Supplementary Fig. S1b, c). We then fed the mice with the methionine-choline deficient (MCD) diet to induce liver fibrosis. Sirius Red staining, a classic histochemical method to visualize collagen fibers, revealed a worsened level of liver fibrosis in the ovariectomized mice challenged by the MCD diet (Supplementary Fig. S1c, d). Moreover, the immunohistochemical assessment of alpha-smooth muscle actin (α-SMA), a cellular marker for activated HSCs, showed enhanced accumulation in the livers of those ovariectomized mice (Supplementary Fig. S1e, f). In addition, activated HSCs express the tissue inhibitor of metalloproteinase 1 (TIMP1) to facilitate the deposition of extracellular matrix by blocking the activity of matrix metalloproteinases. Accordingly, TIMP1 protein levels markedly increased in the livers of the MCD-fed ovariectomized mice, as examined by immunofluorescence staining (Supplementary Fig. S1g, h). Moreover, studies with single-cell RNA sequencing (scRNA-seq) have identified several collagen genes that are highly expressed by HSCs during liver fibrosis, e.g., *Col1a1*, *Col1a2*, and *Col3a1*^9^. mRNA levels of these fibrosis-related genes became significantly elevated in the livers of ovariectomized mice fed with the MCD diet, as examined by quantitative PCR (qPCR) (Supplementary Fig. S1i). These results indicated that sex hormone depletion in adult female mice exaggerated liver fibrosis.

We next tested whether estrogen would be sufficient to enact the resistance to liver fibrosis. Bilateral castration and 17β-estradiol hormone replacement were conducted in adult C57BL/6 wild-type male mice (Supplementary Fig. S2a). Importantly, this approach of hormone replacement did not affect liver steatosis or fibrosis in the experimental timeframe under the NCD condition (Supplementary Fig. S2b, c). On the other hand, the 17β-estradiol treatment strongly reduced the deposition of collagen fibers in the liver tissues of MCD-fed castrated mice (Supplementary Fig. S2c, d). Meanwhile, α-SMA accumulation became suppressed in the livers of those mice (Supplementary Fig. S2e, f). Moreover, TIMP1 expression was inhibited by the 17β-estradiol treatment in MCD-fed mice (Supplementary Fig. S2g, h). In addition, key fibrosis-related genes significantly decreased in the liver tissues of MCD-fed castrated mice (Supplementary Fig. S2i). These results demonstrated that estrogen signaling would be sufficient to mitigate liver fibrosis.

We sought to investigate the molecular mechanism of estrogen signaling in HSCs. HSCs were isolated from human liver tissues (patient information in Supplementary Table S1) by fluorescence-activated cell sorting (FACS), taking advantage of the autofluorescence property of retinol at a wavelength of 405 nm. Human HSCs, which were defined as retinol^+^ CD45^–^ (Supplementary Fig. S3a), were profiled by RNA sequencing (RNA-seq). Notably, previous research in the field reported that HSCs express the estrogen receptor 2 (ESR2, also known as ERβ) for estrogen signaling^10,11^. However, our RNA-seq analyses revealed that HSCs from the liver tissues of both male and female patients predominantly express *ESR1* (also known as ERα) but not *ESR2* (Fig. 1a). In addition, recent studies suggested that human HSCs might express the G protein-coupled estrogen receptor (GPER)^12^. However, our RNA-seq data showed an almost undetectable level of *GPER* in male or female human HSCs (Fig. 1a). In addition, androgen receptor (*AR*) was not expressed in human HSCs (Fig. 1a). We verified this specific expression of ESR1 in human HSCs by immunofluorescence co-staining of ESR1 and α-SMA, unequivocally identifying the ESR1-positive nuclei of α-SMA-positive HSCs in male and female human liver tissues (Fig. 1b; Supplementary Fig. S3c, e and Table S1). In contrast, while ESR2-positive cells appeared in those human liver tissues, anti-ESR2 immunofluorescence signals did not overlap with α-SMA-positive HSCs (Fig. 1c; Supplementary Fig. S3d, e).

**Fig. 1:**
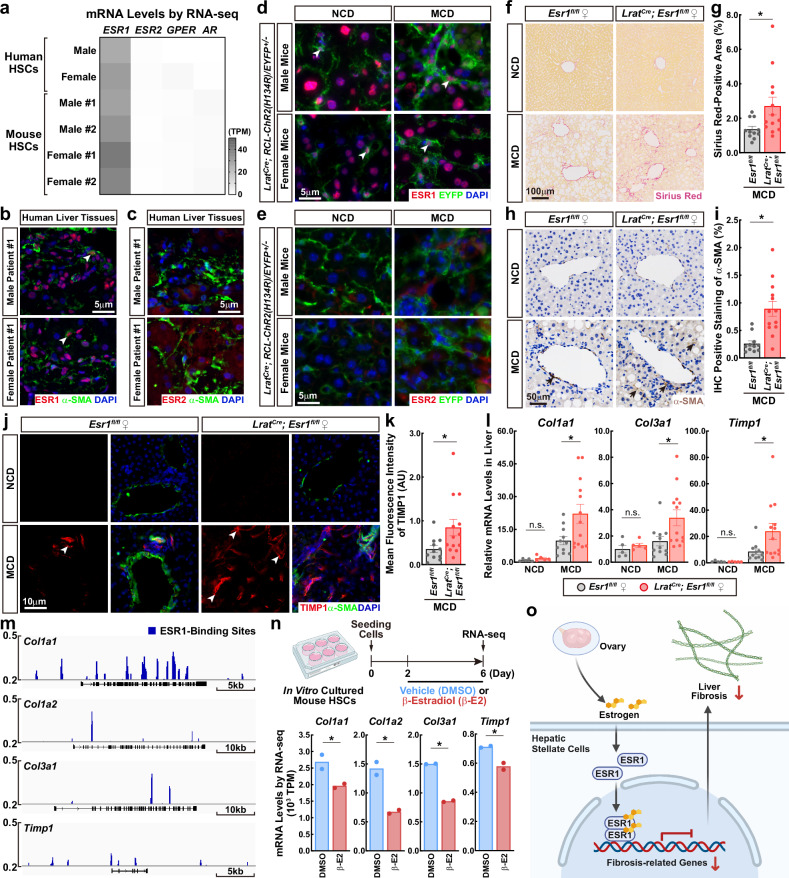
ESR1 signaling in HSCs designates resistance to liver fibrosis. **a** mRNA levels of the indicated receptors for sex hormones in human HSCs (male or female patients; information in Supplementary Table S1) and mouse HSCs (male or female mice; *n* = 2 for each sex) were profiled by RNA-seq. **b**, **c** Paraffin sections of the liver tissue samples of male or female patients (information in Supplementary Table S1) were examined by the immunofluorescence co-staining of α-SMA with ESR1 (**b**) or ESR2 (**c**). White arrowheads exemplify the ESR1-positive nuclei of α-SMA-positive HSCs. **d**, **e** *Lrat*^*Cre*^*;RCL-ChR2(H134R)/EYFP*^*+/–*^ male or female mice were fed with the NCD or MCD diet to induce liver fibrosis. Cryosections of the liver tissues were examined by the immunofluorescence co-staining of EYFP with ESR1 (**d**) or ESR2 (**e**). White arrowheads exemplify the ESR1-positive nuclei of EYFP-positive HSCs. **f**–**l** *Lrat*^*Cre*^*;Esr1*^*fl/fl*^ or control *Esr1*^*fl/fl*^ female littermates were fed with the NCD or MCD diet to induce liver fibrosis. Paraffin sections of the liver tissues were assessed by histochemistry or immunohistochemistry (**f**–**i**). Representative images of Sirius Red staining (**f**) and quantification of the percentage (%) of Sirius Red-positive area (**g**). Representative images of anti-α-SMA immunohistochemistry (**h**, black arrows exemplify anti-α-SMA signals) and quantification of the percentage (%) of α-SMA-positive area (**i**). Data are presented as mean ± SEM; *P < 0.05 (Student’s *t*-test). Cryosections of the liver tissues were examined by the immunofluorescence co-staining of TIMP1 and α-SMA (**j**, **k**). Representative images were shown (**j**). White arrowheads exemplify anti-TIMP1 signals. The mean fluorescence intensity of anti-TIMP1 signals was quantified (**k**). Data are presented as mean ± SEM; *P < 0.05 (Student’s *t*-test). mRNA levels of fibrosis-related genes in the liver tissues were examined by qPCR (**l**). Data are presented as mean ± SEM; *P < 0.05, n.s. not significant (two-way ANOVA test). *n* = 5 for control *Esr1*^*fl/fl*^ or *Lrat*^*Cre*^*;Esr1*^*fl/fl*^ mice under the NCD condition; *n* = 11 for control *Esr1*^*fl/fl*^ mice and *n* = 13 for *Lrat*^*Cre*^*;Esr1*^*fl/fl*^ mice under the MCD condition. **m** *Lrat*^*Cre*^*;RCL-ChR2(H134R)/EYFP*^*+/–*^ female mice were subjected to liver fibrosis. HSCs were then FACS-sorted from the liver tissues and processed for anti-ESR1 ChIP-seq. Genomic browser tracks of ESR1-binding sites at the loci of fibrosis-related genes were shown. **n** HSCs were FACS-sorted from the liver tissues of C57BL/6 wild-type female mice for in vitro culture. The cells were treated with the control vehicle (DMSO) or 17β-estradiol (β-E2), and mRNA levels of fibrosis-related genes were profiled by RNA-seq. *n* = 2 for each condition, *q < 0.05. **o** Diagram of ESR1 signaling in HSCs to mitigate liver fibrosis.

In parallel, we FACS-sorted retinol^+^ CD45^–^ HSCs from adult C57BL/6 wild-type mice (Supplementary Fig. S3b). RNA-seq profiling showed that reminiscent of the observation with human HSCs, both male and female mouse HSCs express *Esr1* but not *Esr2* or *Gper* (Fig. 1a). We further performed the qPCR analyses of male and female mouse HSCs, which confirmed their specific expression of *Esr1* (Supplementary Fig. S3f). In addition, we analyzed the published scRNA-seq datasets GSE136103^13^ and GSE137720^9^, which contain the non-parenchymal cells of adult C57BL/6 wild-type male mice under healthy or fibrotic conditions. Within those pooled datasets, mouse HSCs could be distinguished from other cell types, e.g., vascular smooth muscle cells and fibroblasts (Supplementary Fig. S3g). Of importance, at this single-cell resolution, mouse HSCs primarily expressed *Esr1* in both healthy and fibrotic conditions (Supplementary Fig. S3h). Notably, HSCs decrease their retinol storage upon activation^2^, which may pose an intrinsic limitation of the FACS-based approach for their isolation. We circumvented this issue by exploiting the *Lrat*^*Cre*^ mouse line that drives the Cre recombinase in HSCs. *Lrat*^*Cre*^*;RCL-ChR2(H134R)/EYFP*^*+/–*^ male and female mice were fed with the NCD or MCD diet to induce liver fibrosis. Immunofluorescence co-staining clearly identified the ESR1-positive nuclei of EYFP-positive HSCs in the liver tissues of those NCD-fed or MCD-fed male and female mice (Fig. 1d), but there were no detectable ESR2-positive HSCs (Fig. 1e). Together, these results demonstrated that for both sexes, human and mouse HSCs primarily express ESR1.

We went on to examine the functional relevance of ESR1 signaling in HSCs. To achieve the HSC-specific deletion of *Esr1*, we generated *Lrat*^*Cre*^*;Esr1*^*fl/fl*^ mice. The qPCR analyses showed the complete loss of *Esr1* mRNAs in the HSCs of *Lrat*^*Cre*^*;Esr1*^*fl/fl*^ mice compared to control *Esr1*^*fl/fl*^ mice (Supplementary Fig. S4a), validating the efficiency of this genetic approach. The genetic blockage of ESR1 signaling in HSCs did not cause liver steatosis or fibrosis in the NCD-fed female mice (Fig. 1f; Supplementary Fig. S4b). However, the deposition of collagen fibers following the MCD diet challenge significantly worsened in the livers of *Lrat*^*Cre*^*;Esr1*^*fl/fl*^ female mice (Fig. 1f, g). Further, the liver accumulation of α-SMA (Fig. 1h, i) or TIMP1 (Fig. 1j, k) was elevated in these mice. In addition, the expression of fibrosis-related genes markedly increased in the liver tissues of MCD-fed *Lrat*^*Cre*^*;Esr1*^*fl/fl*^ female mice (Fig. 1l; Supplementary Fig S4c). These results supported that ESR1 signaling in HSCs could protect against liver fibrosis.

We finally looked into the molecular mechanism of ESR1 signaling in HSCs. It has been well documented that upon the estrogen ligand engagement, ESR1 can regulate the expression of target genes whose promoters or enhancer regions contain the specific sequence of estrogen-responsive element (ERE). Therefore, we profiled the ESR1-binding sites in the genome of mouse HSCs by ChIP-seq. EYFP-positive HSCs were FACS-sorted from the *Lrat*^*Cre*^*;RCL-ChR2(H134R)/EYFP*^*+/–*^ female mice subjected to liver fibrosis and then processed for anti-ESR1 ChIP-seq (Supplementary Fig. S5a). Approximately 50% of identified ESR1-binding sites were located in gene regions, including the promoters, untranslated regions, exons, and introns, while others resided in distal intergenic regions (Supplementary Fig. S5b). As expected, de novo motif enrichment analysis showed that ERE was the most enriched motif within those ESR1-binding sites, validating the success of the ChIP-seq procedure (Supplementary Fig. S5c). Notably, the binding motifs of other nuclear receptors were also identified (Supplementary Fig. S5c), e.g., nuclear receptor subfamily 2 group F member 6 (NR2F6, also known as EAR2) and nuclear receptor subfamily 2 group F member 2 (NR2F2, also known as COUP-TFII). This observation revealed the potential genome-wide cooperative action of these nuclear receptors with ESR1. Of importance, we identified the ESR1-binding sites at the loci of key fibrosis-related genes, e.g., *Col1a1*, *Col1a2*, *Col3a1*, and *Timp1* (Fig. 1m), implicating their transcriptional modulation by ESR1. Indeed, the 17β-estradiol treatment of in vitro cultured mouse HSCs effectively suppressed the expression of these genes, as assessed by RNA-seq profiling (Fig. 1n). Meanwhile, we processed *Lrat*^*Cre*^*;RCL-ChR2(H134R)/EYFP*^*+/–*^ mouse HSCs for additional ChIP-seq analyses of the classic histone modifications for transcriptional activity, i.e., H3K27ac and H3K4me1, and the key histone modification for transcriptional silencing, i.e., H3K9me3. The landscapes of these histone markers on *Col1a1*, *Col1a2*, *Col3a1*, and *Timp1* were overall comparable in the HSCs from male or female mice subjected to liver fibrosis (Supplementary Fig. S6a–d), suggesting that the ESR1-mediated transcriptional inhibition would not involve a global alteration of the epigenetic status of these gene loci.

In sum, we have elucidated a critical aspect of ESR1 signaling in HSCs designating resistance to liver fibrosis (Fig. 1o). Future research will help delineate the detailed mechanism underlying the ESR1-mediated transcriptional suppression of fibrosis-related genes, which may act via interactions with other transcriptional factors. It was recently reported that ESR1 could control the expression of the patatin-like phospholipase domain-containing 3 (PNPLA3) I148M variant in human hepatocytes, thus influencing the women’s susceptibility to MASLD^14^. Moreover, previous research has suggested the involvement of mechanotransduction in liver fibrosis^15^. Whether ESR1 signaling may influence the fibrogenic response of HSCs to such mechanical cues warrants more examination. Our in-depth understanding of the sexual dimorphism of liver fibrosis and its pathophysiological mechanisms will lead to better stratification of patients for diagnostic and therapeutic benefits.

## Supplementary information

Supplementary information
