# Supplementary material for: Estrogen receptor 1 signaling in hepatic stellate cells designates resistance to liver fibrosis
Source: Cell Discov. 2025 Apr 15;11:37. doi: 10.1038/s41421-025-00783-3 (PMC12000375; doi:10.1038/s41421-025-00783-3)
Supplement: Supplementary file 1 — Supplementary information [file 41421_2025_783_MOESM1_ESM.pdf]

Supplementary Materials and Methods

Human Liver Tissues

Human liver tissues were collected in compliance with the protocols approved by the Institutional Ethics Committee of Peking University People's Hospital, and informed consent was signed by each involved patient. All the patients were tested negative for the infection of Hepatitis B or Hepatitis C virus. Normal liver tissues were obtained during the hepatectomy for liver diseases (patient information in Supplementary Table S1).

For the fluorescence-activated cell sorting (FACS) of human hepatic stellate cells (HSCs), the procedure was modified from the published protocol [1]. Fresh liver tissues were immediately perfused through main blood vessels with 20ml of the Perfusion Solution (99.6mg/l $NaH_2PO_4 \cdot 2H_2O$, 242.15mg/l $Na_2HPO_4 \cdot 12H_2O$, 350mg/l $NaHCO_3$, 2.38g/l HEPES, 8.0g/l NaCl, 400mg/l KCl, 900mg/l glucose, 190mg/l EGTA, pH 7.35~7.45), 30ml of the Digestion Solution (99.6mg/l $NaH_2PO_4 \cdot 2H_2O$, 242.15mg/l $Na_2HPO_4 \cdot 12H_2O$, 350mg/l $NaHCO_3$, 2.38g/l HEPES, 8.0g/l NaCl, 400mg/l KCl, 560mg/l $CaCl_2 \cdot 2H_2O$, pH 7.35~7.45, pre-warmed to 37°C) containing 0.5mg/ml pronase (Sigma, #P5147), and 40ml of the Digestion Solution containing 0.625mg/ml collagenase II (Biosharp, #BS164). The liver tissues were then minced into small pieces and incubated at 37°C for 15min in the Digestion Solution containing 0.625mg/ml collagenase II and 75U/ml DNaseI (Sigma, #D5025). The digested samples were mashed through 70-μm cell strainers and centrifuged at 50*g* for 3min at 4°C to remove tissue debris and hepatocytes, and the supernatant containing non-parenchymal cells was collected and further centrifuged at 580*g* for 10min at 4°C. The cell pellets were washed with Gey's

Balanced Salt Solution (GBSS; 121.92mg/l $Na_2HPO_4 \cdot 12H_2O$, 30mg/l KH_2PO_4, 227mg/l $NaHCO_3$, 8.0g/l NaCl, 370mg/l KCl, 210mg/l $MgCl_2 \cdot 6H_2O$, 70mg/l $MgSO_4 \cdot 7H_2O$, 225mg/l $CaCl_2 \cdot 2H_2O$, 991mg/l glucose, pH 7.35~7.45) and centrifuged again at 580*g* for 10min at 4°C. The cell pellets were resuspended in GBSS containing a final concentration of 10% (w/v) Nycodenz (Serumwerk Bernburg AG, #18003). 10ml of cell suspension was loaded into each 15ml Falcon tube and gently overlaid with 1.5ml GBSS. The cell preparations were centrifuged at 1,380*g* for 17min at 4°C. The resulting cell layers between the two fractions were collected and stained with FITC anti-human CD45 (BioLegend, #304054) and 7-AAD. The stained non-parenchymal cells were sorted on BD Aria Fusion, and HSCs were identified as retinol (405nm autofluorescence)$^{+}$ CD45^{-}, and the FACS data were processed by FlowJo (https://www.flowjo.com).

For the immunofluorescence staining, human liver tissues were fixed in phosphate-buffered saline (PBS) containing 3.7% (w/v) paraformaldehyde (PFA) at room temperature for 4h. The tissues were then dehydrated in 75% ethanol (diluted in H_2O) for 2h, 85% ethanol for 2h, 90% ethanol for 2h, 100% ethanol for 45min, a mixture (v:v = 1:1) of ethanol and dimethylbenzene for 8min, dimethylbenzene for 8min twice, and paraffin for 1h three times. The paraffin-embedded tissues were sectioned at 4-µm thickness. The sections were rehydrated in dimethylbenzene for 15min twice, 100% ethanol for 5min twice, 75% ethanol for 5min, and sterile water for 5min twice. The sections were then incubated in the citrate antigen retrieval solution (Solarbio, #C1032) at 95°C for 8min, followed by 3% (w/v) hydrogen peroxide solution for 20min. The sections were blocked with PBS containing 3% bovine serum albumin (BSA; Solarbio, #A8010) and immunostained with the intended primary antibodies, including rabbit anti-ESR1

(Millipore, #06-935), rabbit anti-ESR2 (Invitrogen, #PA1-311), and goat anti-α-SMA (Novus, #NB300-978). The sections were finally stained with the corresponding Alexa Fluor-conjugated secondary antibodies, and fluorescence images were scanned by the Olympus SLIDEVIEW VS200.

Mouse Information and Procedures

All the experimental procedures in mice were performed in compliance with the protocol approved by the Institutional Animal Care and Use Committee (IACUC) of Peking University. Mice were maintained on the 12-hr/12-hr light/dark cycle (light period 7:00 am ~ 7:00 pm), with the normal chow diet (NCD) and water available *ad libitum*. The mice used in the experiments were 8 ~ 10 weeks old unless otherwise specified, and the sex of mice for each experiment was specified in the figure legends.

$Lrat^{Cre}$ (Cyagen, #C001205), $Esr1^{fl/fl}$ (Cyagen, #S-CKO-02261), and $RCL\text{-}ChR2(H134R)/EYFP^{+/+}$ (Jackson Laboratory, #012569) were purchased and in-house bred to generate $Lrat^{Cre};Esr1^{fl/fl}$ or $Lrat^{Cre};RCL\text{-}ChR2(H134R)/EYFP^{+/-}$ mice. All the above mouse lines were on the C57BL/6 background. C57BL/6 wild-type mice were purchased from Charles River International.

For the model of liver fibrosis, the mice of indicated conditions were fed with the methionine-choline deficient (MCD) diet (Medicience, #MD12052) for 8 weeks.

For the bilateral ovariectomy, 6-week-old C57BL/6 wild-type female mice were anesthetized with 3% isoflurane, and the back skin was shaved and prepared with iodine and alcohol. The skin and peritoneum incisions were made below the 13th-rib level on both sides to expose ovaries in the peritoneal cavity. The oviduct and blood vessels

supplying each ovary were ligated with a 5/0 suture thread before the ovaries on both sides were resected. Finally, the incisions on the peritoneum and the skin were sutured. Sham surgery included all the steps except the ligation and removal of ovaries. The mice were then subjected to the MCD diet 2 weeks after ovariectomy.

For the bilateral castration and 17β-estradiol hormone replacement, 4-week-old C57BL/6 wild-type male mice were anesthetized with 3% isoflurane. The skin region of the scrotum was shaved and prepared with iodine and alcohol, and a midline incision was made. The testes, vas deferens, and the attached connective tissues on both sides were pulled out. After ligating the vas deferens and blood vessels supplying each testis with a 5/0 suture thread, the testes on both sides were removed. The skin incision was then closed by wound clips (Fine Science Tools). At 3 weeks post-castration, the mice were anesthetized again with 3% isoflurane, and the back skin was shaved and prepared with iodine and alcohol. An incision was made along the skin region of the lower left back, and a 17β-estradiol extended-release pellet (Shinnobio, #XLME-90-B; hormone delivery of 3μg per day) was subcutaneously implanted for each mouse. Finally, the skin incision was closed by a suture. The mice were then subjected to the MCD diet 1 week after receiving the 17β-estradiol replacement.

Histology of Mouse Liver Tissues

The mice of indicated conditions were perfused with 20ml PBS, followed by 20 ml PBS containing 3.7% (w/v) PFA. The liver tissues were dissected and post-fixed in PBS / 3.7% PFA at room temperature for 4h. For the cryosectioning, the tissues were preserved in PBS containing 30% (w/v) sucrose at 4°C for 24h and then embedded in the

optimal cutting temperature compound (OCT; Tissue-Tek, #4583). 10-μm cryosections were blocked with PBS / 3% BSA and immunostained with the intended primary antibodies, including rabbit anti-ESR1 (Millipore, #06-935), rabbit anti-ESR2 (Invitrogen, #PA1-311), goat anti-α-SMA (Novus, #NB300-978), rabbit anti-TIMP1 (Bioss, #BS-0415R), and chicken anti-GFP antibody (Aves Labs, #GFP-1010). The sections were then stained with the corresponding Alexa Fluor-conjugated secondary antibodies, and fluorescence images were scanned by the Olympus SLIDEVIEW VS200. The mean fluorescence intensity of anti-TIMP1 signals was quantified in ImageJ (https://imagej.net/ij).

For the paraffin sectioning, the liver tissues were dehydrated in 75% ethanol for 2h, 85% ethanol for 2h, 90% ethanol for 2h, 100% ethanol for 45min, a mixture (v:v = 1:1) of ethanol and dimethylbenzene for 8min, dimethylbenzene for 8min twice, and paraffin for 1h three times. 4-μm paraffin sections were processed for hematoxylin and eosin (H&E) staining or Sirius Red staining. For the immunohistochemistry, paraffin sections were dewaxed in dimethylbenzene for 15min three times, 100% ethanol for 5min twice, 85% ethanol for 5min, 75% ethanol for 5min, and sterile water for 5min twice. The sections were then incubated in the citrate antigen retrieval solution at 95°C for 20min, followed by 3% hydrogen peroxide solution for 25min. The sections were blocked with PBS / 3% BSA for 30min and sequentially immunostained with the primary rabbit anti-α-SMA (Abcam, #ab5694) and the secondary horseradish peroxidase (HRP)-conjugated goat anti-rabbit IgG (Genetech, #GK500510A). The sections were then treated with the diaminobenzidine (DAB) substrate (Solarbio, #DA1010) for 20min and finally stained with hematoxylin. All the processed paraffin sections were scanned by the Axio Scan Z1.

The Sirius Red-positive area in each section was quantified in ImageJ. The anti-α-SMA immunohistochemistry signal was measured with the H-DAB model in the ImageJ plugin of the Immunohistochemistry Image Analysis Toolbox.

Quantitative PCR (qPCR)

The liver tissues were freshly dissected from the mice of indicated conditions. Total RNAs were extracted by the RNeasy Mini Kit (Qiagen), reverse-transcribed by the PrimeScript RT Reagent Kit with gDNA Eraser (Takara), and analyzed by the SYBR Green Real-Time PCR Kit (Thermo Fisher Scientific). The primer sequences for the qPCR included *Col1a1* (forward: TTCTCCTGGCAAAGACGGAC; reverse: CGGCCACCATCTTGAGACTT), *Col1a2* (forward: CCCAGAGTGGAACAGCGATT; reverse: ATGAGTTCTTCGCTGGGGTG), *Col3a1* (forward: CATGCATAAATGCCAGCCCC; reverse: CCGGCTGGAAAGAAGTCTGA), *Timp1* (forward: TCTTGGTTCCCTGGCGTACTCT; reverse: GTGAGTGTCACTCTCCAGTTTGC), *Esr1* (forward: TCTGCCAAGGAGACTCGCTACT; reverse: GGTGCATTGGTTTGTAGCTGGAC), *Esr2* (forward: TTAGCCACCCACTGCCAATC; reverse: TCACAGGACCAGACACCGTA), *Gper* (forward: GCCACATAGTCAACCTTGCAGC; reverse: CGTCTTCTGCTCCACATAGAGC), *Ar* (forward: CCTTGGATGGAGAACTACTCCG; reverse: TCCGTAGTGACAGCCAGAAGCT), and *B2m* (forward: CTCGGTGACCCTGGTCTTTC; reverse: GGATTTCAATGTGAGGCGGG). *B2m* mRNA levels were utilized as the internal control.

FACS and *In Vitro* Cultures of Mouse HSCs

For the FACS sorting of mouse HSCs, the procedure was modified from the published protocol [1]. The mice of indicated conditions were anesthetized and perfused via the inferior vena cava with 20ml of the Perfusion Solution, followed by 30ml of the Digestion Solution containing 0.5mg/ml pronase and 40ml of the Digestion Solution containing 0.625mg/ml collagenase II. The liver tissues were dissected out and further incubated in the Digestion Solution containing 0.625mg/ml collagenase II and 75U/ml DNaseI at 37°C for 15min. All the solutions were pre-warmed to 37°C before use. The tissues were mashed through 70-μm cell strainers and centrifuged at 50*g* for 3min at 4°C to remove tissue debris and hepatocytes, and the supernatant containing non-parenchymal cells was collected and further centrifuged at 580*g* for 10min at 4°C. The cell pellets were washed in GBSS and centrifuged again at 580*g* for 10min at 4°C. The cell pellets were resuspended in GBSS containing a final concentration of 10% (w/v) Nycodenz. 10ml of cell suspension was loaded into each 15ml Falcon tube and gently overlaid with 1.5ml GBSS. After the centrifugation at 1,380*g* for 17min at 4°C, the resulting cell layers between the two fractions were collected. The non-parenchymal cells were stained with FITC anti-mouse CD45 (BioLegend, #157608) and 7-AAD and sorted on BD Aria Fusion. Mouse HSCs were identified as retinol (405nm autofluorescence)$^{+}$ CD45^{-}, and the FACS data were processed by FlowJo.

Mouse HSCs were *in vitro* cultured according to the published methods [1,2]. FACS-sorted HSCs from the liver tissues of C57BL/6 wild-type female mice were cultured in the Dulbecco's Modified Eagle's Medium (DMEM; Gibco) containing 10%

fetal bovine serum (FBS; NewZerum, #FBS-UE500), 100U/ml penicillin, and 100μg/ml streptomycin for 2 days. The cells were then changed to fresh medium containing a final concentration of 10μM 17β-estradiol (Sigma, #E8875) or the vehicle dimethyl sulfoxide (DMSO) and further cultured for 4 days.

RNA sequencing (RNA-seq)

Total RNAs of human or mouse HSCs were extracted by the RNeasy Mini Kit and subjected to single-end RNA-seq by the Beijing Genomics Institute. Gene expression levels were normalized as transcripts per million (TPM). Differential gene expression analysis was conducted using DESeq2 (v1.34.0) with thresholds set at q-value < 0.05.

Single-cell RNA sequencing (scRNA-seq)

The published scRNA-seq datasets of the non-parenchymal cells of adult male mice, i.e., GSE136103 [3] and GSE137720 [4], were obtained from the Sequence Read Archive (https://www.ncbi.nlm.nih.gov/sra). GSE136103 contains one dataset (GSM4041174) of the control healthy condition and one dataset (GSM4041175) of the CCl_4-induced model of liver fibrosis. GSE137720 contains two datasets (GSM4085623 and GSM4085626) of the control healthy condition and three datasets (GSM4085624, GSM4085625, and GSM4085627) of the CCl_4-induced model of liver fibrosis. For quality control, we filtered out the cells with <800 or >30000 unique molecular identifiers, <500 or >6000 genes, or >10% mitochondrial reads. We further filtered out the doublets in each dataset through the DoubletFinder (https://github.com/chris-mcginnis-ucsf/DoubletFinder).

We loaded the filtered count matrixes to the CreateSeuratObject function in Seurat (v4.0) (https://github.com/satijalab/seurat) to create Seurat objects, which were then merged into one Seurat object, followed by the log-normalization by the NormalizeData function. The top 2,000 variable genes were identified using the FindVariableFeatures function. Principal component analysis (PCA) was performed using the RunPCA function, and batch effect correction was conducted on the principal components with the Harmony function. Unsupervised clustering was performed using the FindNeighbors and FindClusters functions. For the determination of cell types, we referred to the information provided in the original publications [3,4]. The t-Distributed Stochastic Neighbor Embedding (t-SNE) plots were generated by the DimPlot function.

Chromatin Immunoprecipitation Sequencing (ChIP-seq)

Lrat^{Cre};RCL-ChR2(H134R)/EYFP$^{+/-}$ female mice were subjected to the model of liver fibrosis. EYFP$^+$CD45$^-$ HSCs were FACS-sorted from the liver tissues by the procedure described above. 5×10^6 HSCs were collected in 2ml DMEM containing 10% FBS, 100U/ml penicillin, 100μg/ml streptomycin, and 10μM 17β-estradiol. The cells were fixed with 1% formaldehyde at room temperature for 10min and then quenched with a final concentration of 0.3M glycine for 5min. The fixed cells were incubated in 1ml ice-chilled cell lysis buffer (10mM Tris-HCl, 10mM NaCl, 0.2% NP-40/Igepal, pH 8.0) supplemented with 1:500 protease inhibitor cocktail (Roche) and 1:100 phenylmethylsulfonyl fluoride (PMSF; Sigma) for 20min. After cell lysis, nuclei were pelleted at 1,000*g* for 2min at 4°C and then incubated in 1ml nuclear lysis buffer (50mM Tris-HCl, 10mM NaCl, 1% SDS, pH 8.0) supplemented with 1:500 protease inhibitor

cocktail and 1:100 PMSF for 20min on ice. The nuclei lysate containing chromatin was sonicated on the Qsonica Q800R3 sonicator (80% amplitude, 20s ON/40s OFF for 17min). The sonicated samples were centrifuged at 15,000*g* for 10min at 4°C to remove cell debris, and chromatin in the supernatant was collected.

The chromatin samples were diluted with four volumes of dilution buffer (20mM Tris-HCl, 150mM NaCl, 2mM EDTA, 1% Triton X-100, 0.01% SDS, pH 8.0) supplemented with 1:500 protease inhibitor cocktail and 1:100 PMSF. 50µl of protein A/G agarose beads (Santa Cruz, #sc-2003) were added to each sample for pre-clearing at 4°C for 8h with gentle rotation. Each pre-cleared sample was then incubated with 35µl of protein A/G agarose beads pre-bound with 10µl of ChIP-grade rabbit anti-ESR1 (Abcam, #ab32063), rabbit anti-histone H3 (acetyl-K27) (Abcam, #ab4729), rabbit anti-histone H3 (monomethyl-K4) (Abcam, #ab176877), or rabbit anti-histone H3 (trimethyl-K9) (Abcam, #ab8898) at 4°C overnight. Protein A/G agarose beads were washed on ice once with wash buffer I (20mM Tris-HCl, 50mM NaCl, 2mM EDTA, 1% Triton X-100, 0.1% SDS, pH 8.0), twice with high salt buffer (20mM Tris-HCl, 500mM NaCl, 2mM EDTA, 1% Triton X-100, 0.01% SDS, pH 8.0), once with wash buffer II (10mM Tris-HCl, 250mM LiCl, 1mM EDTA, 1% NP-40, 1% sodium deoxycholate, pH 8.0), and twice with TE buffer (10mM Tris-HCl, 1mM EDTA, pH 8.0). The immunoprecipitated chromatin was eluted from protein A/G agarose beads in 200µl elution buffer (100mM $NaHCO_3$, 1% SDS) at room temperature and reverse-crosslinked in the presence of 30µg/ml proteinase K at 65°C overnight. Each eluted sample was supplemented with 10µl of 3M sodium acetate (pH 5.2) and purified by the QIAquick PCR Purification Kit (Qiagen). The ChIP-seq libraries were constructed using the VAHTS Universal DNA

Library Prep Kit for MGI (Vazyme, #NDM607-02) and sequenced on the MGI DNBSEQ-T7 platform.

The ChIP-seq reads were first filtered with Trim Galore (v0.6.6) and then aligned to the mouse genome assembly mm9 using Bowtie2 (v2.3.5.1). Low-quality reads (MAPQ <30) were filtered out, and reads that aligned to mitochondria random contigs and ENCODE blacklisted regions were removed. In addition, PCR duplicates were removed using Picard (v2.23.3). Valid reads were then normalized using the counts per million (CPM) method by Deeptools (v3.1.3). Enrichment analysis of transcription factor motifs was performed on the narrow peak file using the findMotifsGenome.pl module of HOMER v.4.11 with the parameters "-size 200 -len 8 -mis 2 -S 5". Background sequences were derived from the mm9 genome in the HOMER database. The top motifs from the known matches are visualized by the R package universal motif (v.1.18.1).

Data Availability

The RNA-seq and ChIP-seq datasets of this study were deposited to the Gene Expression Omnibus (https://www.ncbi.nlm.nih.gov/geo/) with the accession number GSE275942.

Statistical Methods

Student's *t*-test (unpaired two-tailed) or ANOVA (two-way with *post hoc* tests) was performed by GraphPad Prism (http://www.graphpad.com/scientific-software/prism). Student's *t*-test was used to compare two groups, and ANOVA was utilized for multiple group comparisons. The sample numbers (n) shown in the figures represent biological

replicates (i.e., patients or mice). The results were reported as mean ± SEM (standard error of the mean), and the statistical information was included in the figure legends where appropriate.

References

1 Mederacke, I., Dapito, D. H., Affo, S., Uchinami, H. & Schwabe, R. F. High-yield and high-purity isolation of hepatic stellate cells from normal and fibrotic mouse livers. *Nat Protoc* **10**, 305-315, doi:10.1038/nprot.2015.017 (2015).

2 Arab, J. P. *et al.* Hepatic stellate cell activation promotes alcohol-induced steatohepatitis through Igfbp3 and SerpinA12. *J Hepatol* **73**, 149-160, doi:10.1016/j.jhep.2020.02.005 (2020).

3 Duan, Y. *et al.* CRIg on liver macrophages clears pathobionts and protects against alcoholic liver disease. *Nat Commun* **12**, 7172, doi:10.1038/s41467-021-27385-3 (2021).

4 Dobie, R. *et al.* Single-Cell Transcriptomics Uncovers Zonation of Function in the Mesenchyme during Liver Fibrosis. *Cell Rep* **29**, 1832-1847 e1838, doi:10.1016/j.celrep.2019.10.024 (2019).

Supplementary Figures

Figure S1

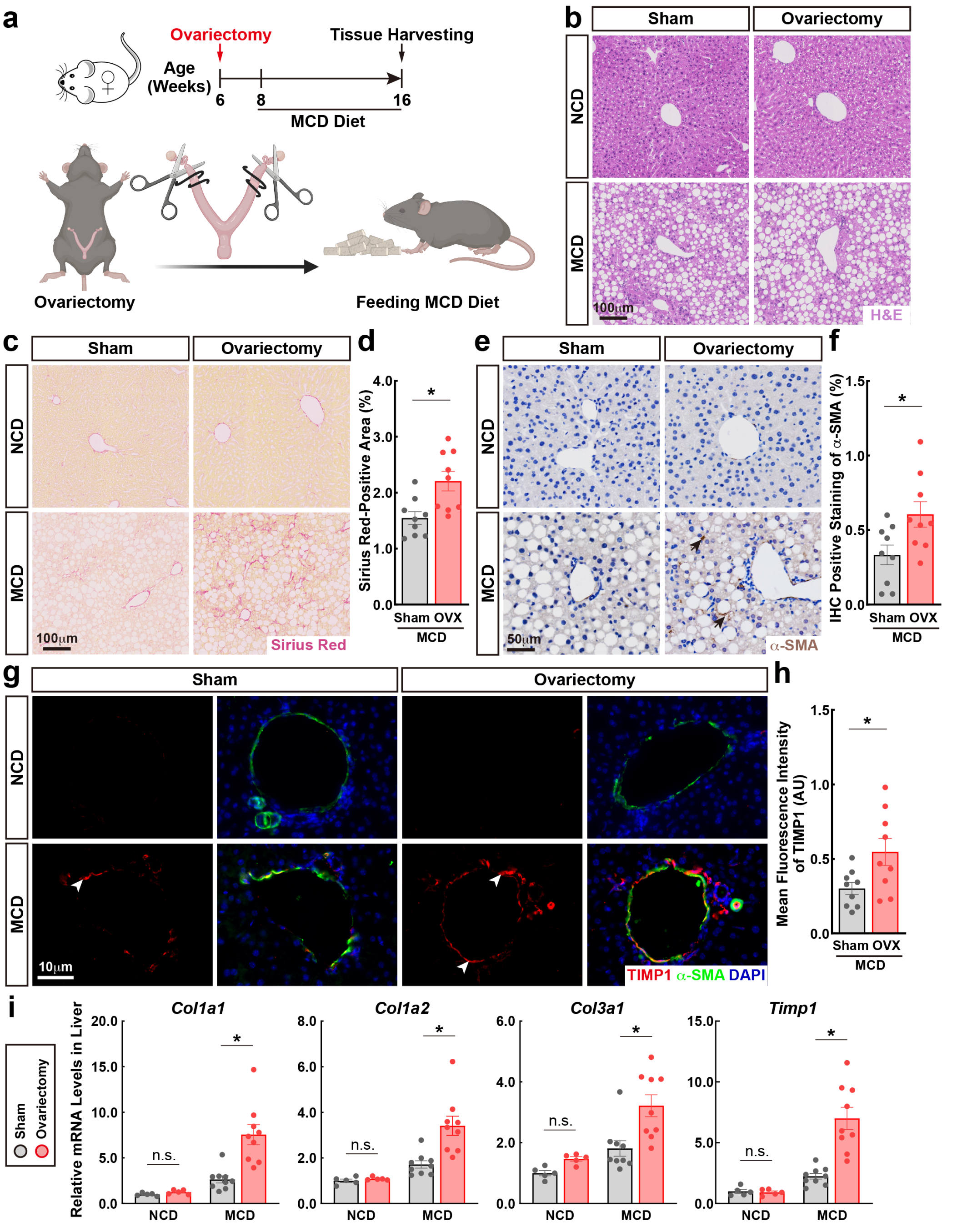

Figure S1. Sex hormone depletion in adult female mice exaggerates the MCD diet-induced liver fibrosis.

6-week-old C57BL/6 wild-type female mice underwent bilateral ovariectomy or sham surgery. The mice were then fed with the normal chow diet (NCD) or methionine-choline deficient (MCD) diet to induce liver fibrosis.

(a) Diagram of the experimental procedure. **(b to f)** Paraffin sections of the liver tissues were assessed by histochemistry or immunohistochemistry. **(b)** Representative images of H&E staining. **(c and d)** Representative images of Sirius Red staining **(c)** and the quantification of the percentage (%) of Sirius Red-positive area **(d)**. **(e and f)** Representative images of anti-α-SMA immunohistochemistry **(e**, black arrows exemplify anti-α-SMA signals**)** and the quantification of the percentage (%) of α-SMA-positive area **(f)**. Mean ± SEM, * $p < 0.05$ (Student's *t*-test). **(g and h)** Cryosections of the liver tissues were examined by the immunofluorescence co-staining of TIMP1 and α-SMA. **(g)** Representative images were shown. White arrowheads exemplify anti-TIMP1 signals. **(h)** The mean fluorescence intensity of anti-TIMP1 signals was quantified. Mean ± SEM, * $p < 0.05$ (Student's *t*-test). **(i)** mRNA levels of fibrosis-related genes in the liver tissues were determined by qPCR. Mean ± SEM, * $p < 0.05$, n.s., not significant (two-way ANOVA test). n = 5 for sham control or ovariectomized mice under the NCD condition; n = 9 for sham control or ovariectomized mice under the MCD condition.

Figure S2

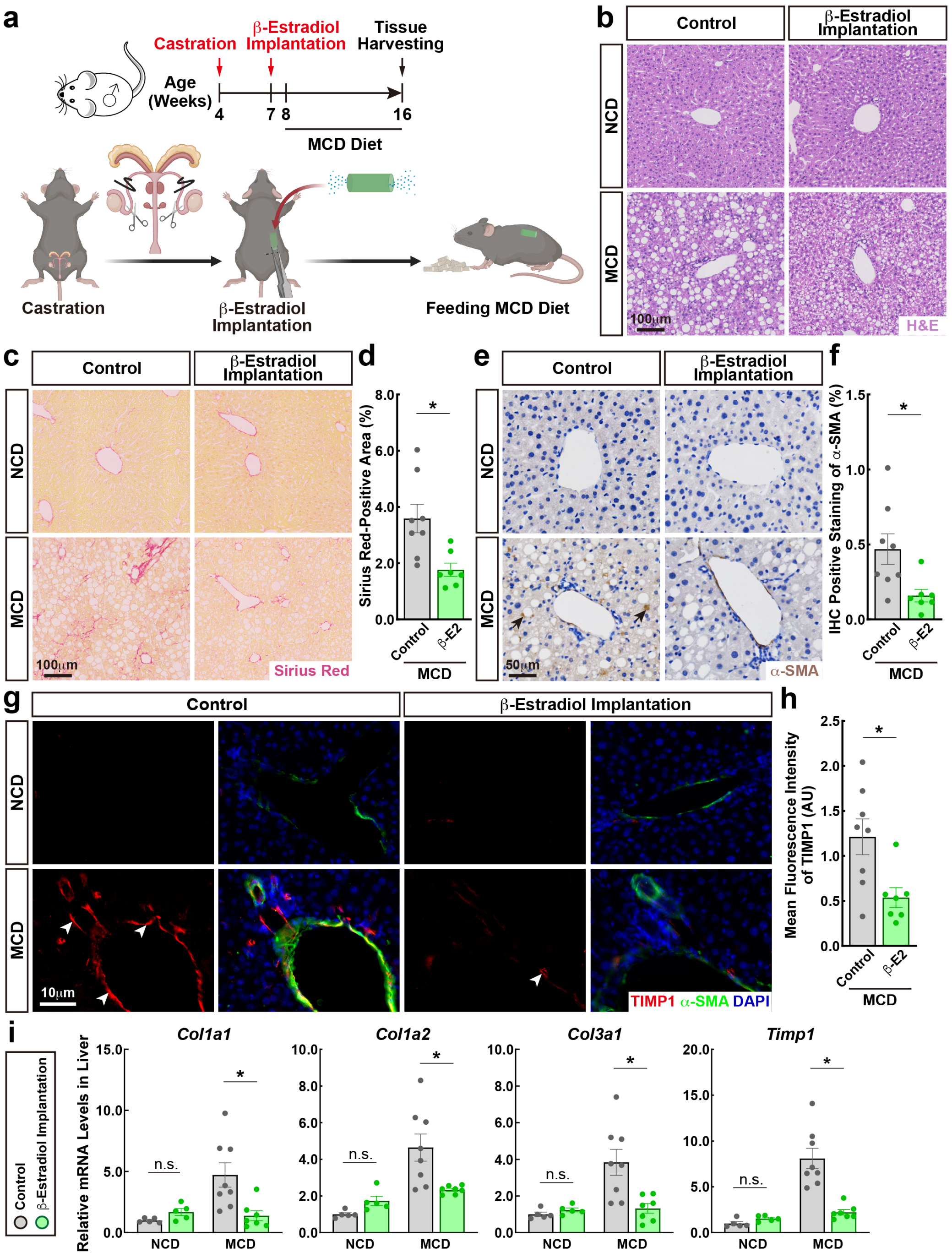

Figure S2. Estrogen replacement in castrated adult male mice is sufficient to mitigate liver fibrosis.

4-week-old C57BL/6 wild-type male mice underwent bilateral castration and 17β-estradiol (β-E2) hormone replacement. The mice then received the normal chow diet (NCD) or methionine-choline deficient (MCD) diet to induce liver fibrosis.

(a) Diagram of the experimental procedure. **(b to f)** Paraffin sections of the liver tissues were assessed by histochemistry or immunohistochemistry. **(b)** Representative images of H&E staining. **(c and d)** Representative images of Sirius Red staining **(c)** and the quantification of the percentage (%) of Sirius Red-positive area **(d)**. **(e and f)** Representative images of anti-α-SMA immunohistochemistry **(e**, black arrows exemplify anti-α-SMA signals**)** and the quantification of the percentage (%) of α-SMA-positive area **(f)**. Mean ± SEM, * $p < 0.05$ (Student's *t*-test). **(g and h)** Cryosections of the liver tissues were examined by the immunofluorescence co-staining of TIMP1 and α-SMA. **(g)** Representative images were shown. White arrowheads exemplify anti-TIMP1 signals. **(h)** The mean fluorescence intensity of anti-TIMP1 signals was quantified. Mean ± SEM, * $p < 0.05$ (Student's *t*-test). **(i)** mRNA levels of fibrosis-related genes in the liver tissues were examined by qPCR. Mean ± SEM, * $p < 0.05$, n.s., not significant (two-way ANOVA test). n = 5 for untreated control or β-E2-treated mice under the NCD condition; n = 8 for untreated control mice and n = 7 for β-E2-treated mice under the MCD condition.

Figure S3

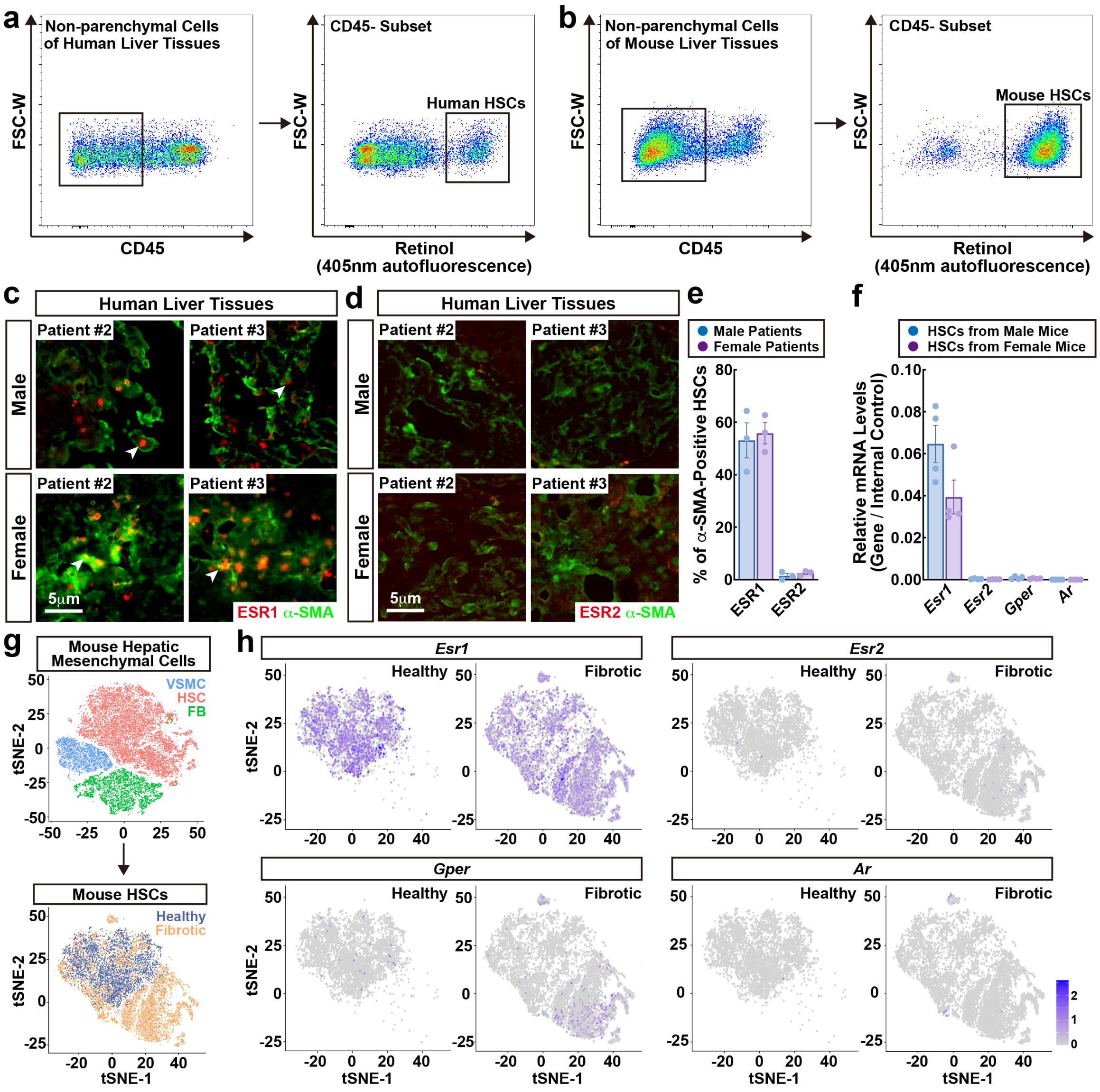

Figure S3. Human and mouse HSCs predominantly express ESR1.

(a and b) Human or mouse HSCs were FACS-sorted from the liver tissues. Representative FACS plots of human HSCs **(a)** or mouse HSCs **(b)** that were identified as retinol (405nm autofluorescence)$^{+}$ CD45^{-}.

(c to e) Paraffin sections of the liver tissue samples of male or female patients

(information in Supplementary Table S1) were examined by the immunofluorescence co-staining of α-SMA with ESR1 **(c)** or ESR2 **(d)**. White arrowheads exemplify the ESR1-positive nuclei of α-SMA-positive HSCs. **(e)** The percentage (%) of ESR1-positive or ESR2-positive HSCs in human liver tissues was quantified. n = 3 for each sex, mean ± SEM.

(f) HSCs were FACS-sorted from the liver tissues of C57BL/6 wild-type male or female mice. mRNA levels of the indicated receptors for sex hormones were examined by qPCR. n = 4 for each sex, mean ± SEM.

(g and h) The published scRNA-seq datasets GSE136103 and GSE137720 of the non-parenchymal cells of C57BL/6 wild-type male mice were analyzed. **(g)** t-SNE plots of the pooled datasets (upper panel) and HSCs in control healthy or fibrotic conditions (lower panel). VSMC, vascular smooth muscle cells; FB, fibroblasts. **(h)** Feature plots of the expression of indicated receptors for sex hormones in mouse HSCs of control healthy or fibrotic conditions.

Figure S4

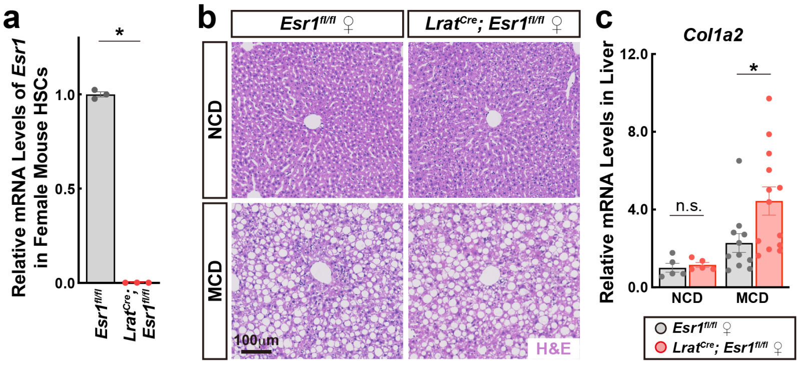

Figure S4. Genetic blockage of ESR1 signaling in HSCs aggregates the model of liver fibrosis.

Lrat^{Cre};*Esr1*$^{fl/fl}$ or control *Esr1*$^{fl/fl}$ female littermates received the normal chow diet (NCD) or methionine-choline deficient (MCD) diet to induce liver fibrosis.

(a) HSCs were FACS-sorted from the liver tissues, and the genetic deletion of *Esr1* in *Lrat*^{Cre};*Esr1*$^{fl/fl}$ HSCs was validated by qPCR. n = 3 for control *Esr1*$^{fl/fl}$ or *Lrat*^{Cre};*Esr1*$^{fl/fl}$ mice, mean ± SEM, * $p < 0.05$ (Student's *t*-test). **(b)** Representative images of H&E staining of the liver tissues. **(c)** *Col1a2* mRNA levels in the liver tissues were determined by qPCR. n = 5 for control *Esr1*$^{fl/fl}$ or *Lrat*^{Cre};*Esr1*$^{fl/fl}$ mice under the NCD condition; n = 11 for control *Esr1*$^{fl/fl}$ mice and n = 13 for *Lrat*^{Cre};*Esr1*$^{fl/fl}$ mice under the MCD condition. Mean ± SEM, * $p < 0.05$, n.s., not significant (two-way ANOVA test).

Figure S5

a

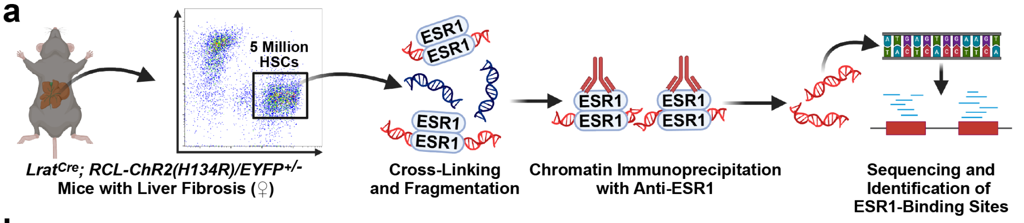

b

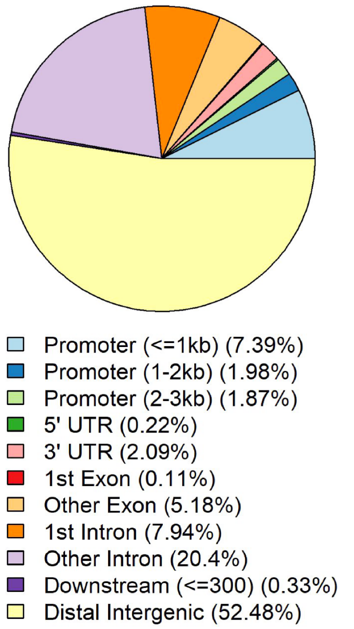

c

Motif	Best Match	Consensus	*p*-value	% of Background Sequence with Motif
	ERE	VAGGTCACNSTGACC	1.00E-131	3.28%
	EAR2	NRBCARRGGTCA	1.00E-21	16.36%
	COUP-TFII	AGRGGTCA	1.00E-20	20.98%
	COUP-TFII	GKBCARAGGTCA	1.00E-15	17.33%
	THRb	TRAGGTCA	1.00E-11	49.13%
	RARa	TTGAMCTTTG	1.00E-10	33.11%
	Reverb	GTRGGTCASTGGGTCA	1.00E-09	1.62%
	Stat3	CTTCCGGGAA	1.00E-06	4.71%
	GRE	VAGRACAKWCTGTYC	1.00E-05	2.32%

Figure S5. ESR1 directly targets key fibrosis-related genes in mouse HSCs. *Lrat*^{Cre}*;RCL-ChR2(H134R)/EYFP*$^{+/-}$ female mice were subjected to the model of liver fibrosis. HSCs were then FACS-sorted from the liver tissues and processed for anti-ESR1 chromatin immunoprecipitation sequencing (ChIP-seq).

(a) Diagram of the experimental procedure. **(b)** Summary of the genomic distribution of ESR1-binding sites in mouse HSCs. **(c)** *De novo* motif enrichment analysis of the motifs identified in ESR1-binding sites.

Figure S6

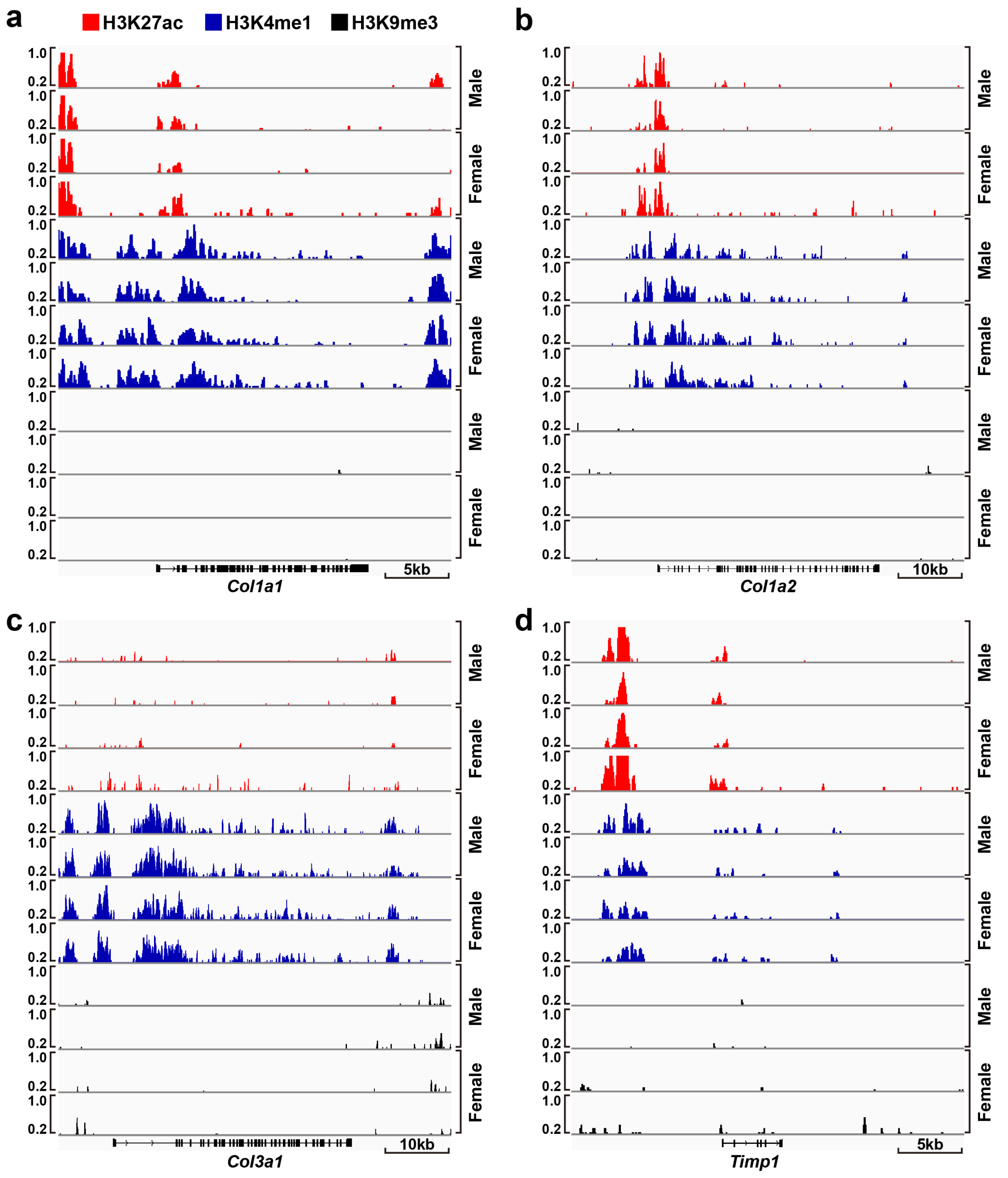

Figure S6. Epigenetic landscapes of fibrosis-related genes in mouse HSCs. *Lrat*^{Cre}*;RCL-ChR2(H134R)/EYFP*$^{+/-}$ male or female mice were subjected to the model of liver fibrosis. HSCs were then FACS-sorted from the liver tissues and processed for anti-H3K27ac, anti-H3K4me1, or anti-H3K9me3 ChIP-seq analyses. n = 2 for each sex.

(a-d) Genomic browser tracks of the landscapes of H3K27ac, H3K4me1, and H3K9me3 at the gene locus of *Col1a1* **(a)**, *Col1a2* **(b)**, *Col3a1* **(c)**, or *Timp1* **(d)** were shown.

Supplementary Table

Table S1. Information of human liver tissues.

Patient#	Age	Disease	Application
Male Patient #1	57	Hepatocellular Carcinoma	Immunofluorescence Staining
Male Patient #2	45	Hepatocellular Carcinoma	Immunofluorescence Staining
Male Patient #3	49	Liver Hemangioma	Immunofluorescence Staining
Male Patient #4	76	Hepatocellular Carcinoma	RNA-seq of HSCs
Female Patient #1	33	Liver Cysts	Immunofluorescence Staining
Female Patient #2	62	Hepatocellular Carcinoma	Immunofluorescence Staining
Female Patient #3	50	Liver Hemangioma	Immunofluorescence Staining
Female Patient #4	70	Cholangiocarcinoma	RNA-seq of HSCs
